# Imaging Findings of Primary Splenic Lymphoma: A Review of 17 Cases in Which Diagnosis Was Made at Splenectomy

**DOI:** 10.1371/journal.pone.0080264

**Published:** 2013-11-21

**Authors:** Meng Li, Li Zhang, Ning Wu, Wenting Huang, Ning Lv

**Affiliations:** 1 Department of Diagnostic Radiology, Cancer Hospital, Chinese Academy of Medical Sciences, Peking Union Medical College, Beijing, China; 2 PET-CT Center, Cancer Hospital, Chinese Academy of Medical Sciences, Peking Union Medical College, Beijing, China; 3 Department of Pathology, Cancer Hospital, Chinese Academy of Medical Sciences, Peking Union Medical College, Beijing, China; University of North Carolina at Chapel Hill, United States of America

## Abstract

**Purpose:**

This study sought to characterize the imaging features of primary splenic lymphoma (PSL).

**Materials and Methods:**

Pathological and imaging data from 17 patients with primary splenic lymphoma initially diagnosed at splenectomy were retrospectively analyzed. Pretreatment computed tomography (CT) imaging was available for 16 patients, and magnetic resonance imaging (MRI) data were available for 4 patients. Splenic lymphoma imaging data were categorized based on the gross pathological presentation in the following manner: type 1, homogeneous enlargement; type 2, miliary nodules; type 3, multifocal masses of varying size; and type 4, solitary large mass.

**Results:**

Of the 17 patients with PSL, 16 cases were non-Hodgkin lymphoma, and of these, 9 cases were diffuse large B cell lymphomas (DLBCL) and 4 cases were splenic marginal zone B-cell lymphoma (SMZL). Imaging showed the following types of PSL presentation: 1 case of type 1, 0 cases of type 2, 4 cases of type 3, and 12 cases of type 4. There was evidence of necrosis in 12 cases (70.6%), and there was evidence of mild enhancement in enhanced CT in 14 cases and in enhanced MRI in 3 cases. Prior to surgery, PSL was considered possible in 8 patients.

**Conclusion:**

The most frequent histological subtype was DLBCL, followed by SMZL. In both CT and MRI, PSL generally presents as a solitary mass or masses rather than as splenomegaly. In addition, necrosis and mild enhancement are commonly observed, and splenectomy may be required to confirm the diagnosis.

## Introduction

Because of its role in filtering the blood, the spleen is commonly involved in hematologic malignancies, of which lymphoma is the most common [1]. Splenic lymphoma is often a manifestation of the diffuse dissemination characteristic of Hodgkins and non-Hodgkins lymphoma, the diagnosis of which is generally made via biopsy of peripheral lymph nodes or bone marrow. However, primary splenic lymphoma (PSL) is quite rare, with a reported incidence of less than 1% [2], and the initial diagnosis of this condition is generally established at the time of splenectomy.

In this report, we present 17 cases of malignant lymphoma with prominent splenic involvement in which the original diagnosis was made at splenectomy. In this case series, we emphasize the accompanying imaging findings for these patients, including computed tomography (CT) and magnetic resonance imaging (MRI) results.

## Materials and Methods

### Patient enrollment

Our institutional ethics committee (Ethics Committee of Cancer Institute and Hospital, Chinese Academy of Medical Sciences) approved this retrospective study. Our institutional ethics committee specifically approved the absence of informed consent because of the retrospective nature of the study and because the data were analyzed anonymously.

Between January 2005 and March 2012, the clinical, pathological, and imaging (including CT and/or MRI) records of all lymphoma patients (n = 3,354) for whom the initial histopathologic diagnosis of lymphoma was made at splenectomy and who were treated at the Cancer Hospital of the Chinese Academy of Medical Science & Peking Union Medical College were reviewed retrospectively. All patients included in this study had no previously established lymph node or bone marrow diagnosis of lymphoma. Splenectomy was performed to determine the pathological diagnosis and to offer curative treatment rather than relieve symptomatic splenomegaly or reverse cytopenias. The patients received no chemotherapy or radiation therapy prior to surgery. After exclusion of patients who did not meet the criteria above, a total of 17 patients with primary splenic lymphoma remained. None of these patients had AIDS, and 1 patient had a surgical history of buccal mucosa carcinoma.

### CT equipment and scan parameters

The following CT equipment was used: Toshiba Aquilion 64-MDCT (pitch 1, thickness 5 mm, 120 kV, 380 mA, reconstruction thickness 1 mm, and spacing 0.8 mm); GE Lightspeed 64-VCT (pitch 1, thickness 5 mm, 120 kV, 450 mA, reconstruction thickness 1.25 mm, and spacing 0.8 mm); and GE Lightspeed Ultra 8-MDCT (pitch 1, thickness 5 mm, 120 kV, 350 mA). When contrast-enhanced scanning was performed, the arterial, portal venous, and delayed phases were scanned at 30∼35, 60∼65, and 150∼180 seconds, respectively, after intravenous contrast injection.

### MRI equipment and scan parameters

A GE 1.5/3.0 T MRI scanner and 8US Torsopa body coil were used. Routine MRI plain scans consisted of axial FSPGR T_1_WI (TR 249 ms, TE 2.3 ms, slice thickness 7 mm, slice space 1 mm, NEX 1, FOV 36×32) and FSE T_2_WI (TR 6,667 ms, TE 104 ms, slice thickness 7 mm, slice space 1 mm, NEX 2, FOV 36×32) with fat suppression as well as diffusion weighted imaging (DWI, TR 3,950 ms, TE 65.2 ms, FOV 36×32, slice thickness 7 mm, slice space 1 mm, NEX 2, b value 800). Dynamic contrast-enhanced scanning was performed in fast acquisition mode using multiphase enhanced fast GRE and the following parameters: TR 2.9 ms, TE 1.4 ms, flip angle 15°, NEX 0.7, slice thickness 6 mm, and FOV 36×32.

### Imaging and histopathologic analyses

We analyzed the clinical, pretreatment imaging, and histopathologic data of the included patients. Imaging data were reviewed for splenic appearance and perisplenic involvement on CT/MRI. Histological subtyping was performed according to World Health Organization (WHO) classification systems [3], [4].

We categorized the splenic lymphoma imaging data based on the gross pathological presentation in the following manner: type 1, homogeneous enlargement; type 2, miliary nodules; type 3, multifocal masses of varying size; and type 4, solitary large mass.

PSL patients were grouped into 3 disease stages based on Ahmann's criteria: stage I, patients with a tumor limited to the spleen; stage II, those with splenic hilar node involvement in addition to splenic involvement; and stage III, those with hepatic or lymph node involvement beyond the splenic hilum [2].

## Results

### Clinical presentations

The sample population included 12 males and 5 females with a median age of 54 years and an age range of 32 to 74 years. The clinical presentations included left upper quadrant pain (8 cases), waist pain (2 cases), weight loss (1 case), and fever (1 case). Four patients had splenomegaly or the presence of a palpable abdominal mass. Five patients were without any signs or symptoms but were found to have lymphoma incidentally as a result of physical examination.

### Pathological findings

Of the 17 cases of PSL, 16 cases were non-Hodgkin lymphoma (NHL). Of these NHL cases, there were 9 cases of diffuse large B-cell lymphoma (DLBCL), 4 cases of splenic marginal zone B-cell lymphoma (SMZL), 2 cases of follicular lymphoma (FL), and 1 case of splenic diffuse red pulp small B-cell lymphoma (SDRL). There was only 1 case of nodular lymphocyte-prominent Hodgkin lymphoma (NLPHL) (Table 1).

**Table 1:** Pathological and imaging features of 17 PSLs diagnosed at splenectomy.

Patient	Age (y)	Gender	Subtype of pathology	Imaging modality	Enhancement	Necrosis	Imaging type	Ahmann stage
1	66	Male	DLBCL	CT/MRI	Mild	Y	4	I
2	56	Female	DLBCL	CT	Obvious	Y	4	III
3	45	Female	DLBCL	CT	Mild	Y	4	I
4	36	Male	DLBCL	MRI	Mild	N	4	III
5	74	Male	DLBCL	CT	No contrast scan	Y	4	III
6	61	Female	DLBCL	CT	Mild	Y	3	III
7	47	Male	SMZL	CT	Mild	Y	3	III
8	74	Male	DLBCL	CT	Mild	N	4	I
9	34	Male	FL (Grade III)	CT	Mild	Y	3	III
10	32	Male	SMZL	CT	Mild	N	4	I
11	41	Female	DLBCL	CT/MRI	Mild	Y	4	I
12	43	Male	FL(Grade II)	CT	Mild	Y	4	I
13	70	Male	SDRL	CT/MRI	Mild	N	4	I
14	72	Male	SMZL	CT	Mild	N	1	III
15	57	Male	DLBCL	CT	Mild	Y	4	I
16	51	Female	SMZL	CT	Mild	Y	4	III
17	59	Male	NLPHL	CT	Mild	Y	3	III

DLBCL: diffuse large B cell lymphoma; SMZL: splenic marginal zone B-cell lymphoma; FL: follicular lymphoma; SDRL: splenic diffuse red pulp small B-cell lymphoma; NLPHL: nodular lymphocyte predominant Hodgkin lymphoma.

### Imaging findings

#### CT findings

**Figure 1:**
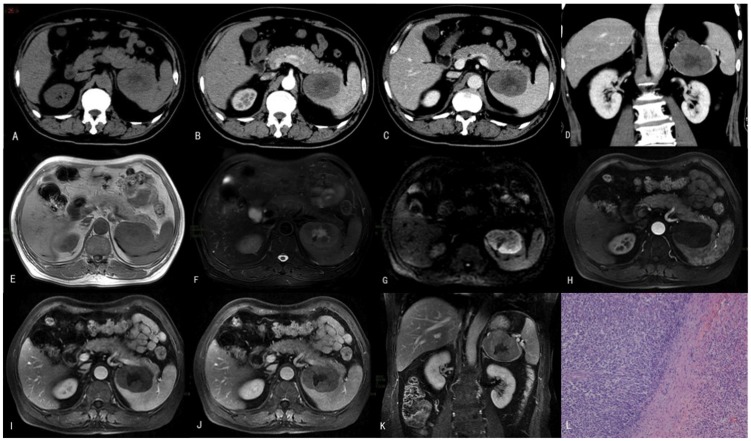
The case of a 66-year-old male patient who had experienced left upper quadrant pain for 1 month is presented. This patient was diagnosed with PSL at splenectomy. (A) A CT plain scan showed a solitary focal mass with faint hypodensity in the spleen. Contrast-enhanced CT showed mild enhancement of the mass in the (B) arterial phase, (C) the portal phase, and (D) the coronal portal phase. The central hypodensity within the mass represents necrosis. MRI plain scans showed a hypointense signal in (E) T_1_WI and a hyperintense signal in (F) T_2_WI and (G) DWI. Enhanced MRI findings for the (H) arterial phase, (I) the portal phase, (J) the delayed phase and (K) the coronal delayed phase demonstrated mild enhancement. (L) The histopathology of the splenic lesion was suggestive of diffuse large B-cell lymphoma (DLBCL) (H&E, 100×).

Sixteen patients underwent CT examinations (10 patients underwent abdomen CT scan and 6 patients underwent abdomen-pelvis CT scan). Of these, 15 patients underwent enhanced CT examination (6 patients received an additional plain CT), and 1 patient underwent plain CT examination alone. For the 7 unenhanced CT scans, there were 4 faint hypodense lesions and 3 isodense lesions observed with respect to the normal splenic parenchyma; however, these lesions produced an enlarged spleen with a lobular contour, suggesting the presence of a tumor. All 16 cases showed hypo-enhancement in comparison to the surrounding splenic parenchyma; of these, 14 cases showed mild enhancement similar to muscle tissue, and 1 case had obvious enhancement comparable to liver tissue. Twelve cases showed evidence of necrosis, which was represented by irregular areas without enhancement in the center of the tumor, and 7 cases received a diagnosis of DLBCL (Figure 1). All 17 patients had chest X-ray film and 3 patients had neck-chest CT prior to surgery. Additionally 9 patients had neck-chest CT and 2 patients had PET-CT exam after surgery when diagnosis of PSL was proved pathologically. The additional imaging did not reveal any additional sites of disease.

#### MRI findings

Four patients underwent abdomen MRI scans prior to surgery. Of these, 3 patients underwent plain and enhanced MRI examinations simultaneously, and 1 patient underwent plain MRI examination only. On T_1_WI, all lesions were hypointense relative to the normal splenic parenchyma. On T_2_WI, the lesions were hyperintense (2 cases), isointense (1 case), or hypointense (1 case). On DWI, all lesions were hyperintense. The lesions demonstrated mild enhancement and were hypointense relative to the clearly enhanced spleen on all 3 contrast-enhanced scans (Figure 1).

#### Perisplenic involvement

Two cases showed evidence of invasion of adjacent organs, including the pancreas and the stomach. Nine patients had splenic hilar and retroperitoneal lymph node involvement. There were no patients with splenic lymph node involvement only.

#### Imaging type and disease stage

CT and/or MRI findings showed 1 case of type 1, 0 cases of type 2, 4 cases of type 3, and 12 cases of type 4. According to the pathological and imaging results, 8 cases were classified as stage I disease, 0 cases as stage II disease, and 9 cases as stage III disease.

### Imaging diagnosis prior to surgery

Prior to surgery, PSL at CT/MRI was considered possible in only 8 patients. Of the 8 patients with stage I disease, only 1 patient was diagnosed with the possibility of PSL at CT/MRI.

## Discussion

### Primary splenic lymphoma, PSL

Cases of malignant lymphoma initially diagnosed at splenectomy without evidence of peripheral disease are very rare. This condition is also referred to as malignant lymphoma with prominent splenic involvement (LPS). At our institution, the incidence of this condition is approximately 0.5% (17/3354), which is slightly lower than that noted in a previous report [2]. This difference may be because all of the patients enrolled in our study had undergone CT and/or MRI examination.

The defining criteria noted in the literature for PSL are ambiguous and vary widely. Dasgupta first defined this condition as confined to the spleen or hilar lymph nodes with no recurrence of disease for at least 6 months after splenectomy [5]. Dachman's report mainly continues this strict definition. In this previous report, extra-splenic involvement was limited by clear direct extension through the capsule to a contiguous structure, and the report did not allow for involvement of peripheral lymph nodes and bone marrow [6]. However, there are very few patients who fulfill these criteria. Many researchers, such as Kehoe, have used a broad definition and have included patients with concurrent minimal involvement of the liver as well as lymph nodes beyond the splenic hilum, as long as the bulk of disease was within the spleen [7]. In our study, all enrolled patients fulfilled the broad definition proposed by Kehoe, while only 8 patients fulfilled the strict definition proposed by Dasgupta.

In fact, PSL based on the strict criteria is equivalent to stage I/II PSL according to the broad definition and represents localized disease. Lymphoma originating in the spleen may spread to other sites, such as abdominal lymph nodes and the liver. Thus, we chose to adopt the broad criteria, which included stage III patients with minimal involvement of abdominal lymph nodes and the liver for whom the bulk of the disease was found in the spleen and for whom splenectomy was necessary for diagnosis and healing. Thus, we speculated that for such patients, lymphoma originated in the spleen.

### Clinical and pathological features

Clinical manifestations depend on various parameters, e.g., the location of the lymphoma, the rate of tumor growth, and the function of the spleen being compromised or displaced by the malignant procedure. According to our results, the most common clinical presentation of PSL is left upper quadrant pain, followed by waist pain, weight loss, and fever. However, these symptoms are nonspecific.

The vast majority of PSL cases consist of NHL [2], [6], [8]. A range of histologic subtypes may initially present with splenic involvement, and the classification of histologic type for such malignancies is quite varied due to the complicated histological appearance of NHL [2], [6]–[9]. In our study, most patients had NHL (94.1%, 16/17), and the most common histological subtype was DLBCL (56.3%, 9/16) followed by SMZL (25.0%, 4/16).

### Imaging features

#### CT findings

Our study showed that the PSL lesions usually presented with hypo-attenuation (4/7) or iso-attenuation (3/7) in plain CT. Significant necrosis may also occur, which can be observed with cross-sectional imaging, particularly in large tumors and in histologically defined large cell lymphomas [10], [11]. In this study, area of necrosis were observed in 70.6% (12/17) of cases, and 77.8% (7/9) of DLBCL cases showed evidence of necrosis.

Ahmann classified the gross pathological appearance of lyphomatous involvement of the spleen (generalized or primary) into the following 4 categories: (1) homogeneous enlargement without masses, (2) miliary masses, (3) 2–10 cm masses, and (4) large solitary mass [2]. The imaging features of splenic lymphomas in CT have been shown to correlate with their macroscopic appearances, and radiologists classify the imaging appearance of spleen lymphoma in a similar manner with very minor differences [6],[12]. In our report, we adopted this previous categorization scheme.

Diffuse, uniform infiltration is the most common pathological form of splenic lymphoma; nevertheless, it presents as normal or only shows splenomegaly on imaging. Although these are the most common imaging findings, splenomegaly is not diagnostically specific because the spleen can be normal in size despite tumor infiltration or may be enlarged without neoplastic involvement due to a reactive process [13], [14]. Because it identifies increased glucose metabolism by tumor cells, PET-CT is superior to CT in evaluating splenic involvement regardless of morphology [15]. However, in our study, 75% (12/16) of splenic lesions manifested as a solitary mass on CT (type 4), indicating that PSL usually presents as a mass rather than as splenomegaly alone. Our result is also consistent with previous reports [6], [16], [17], although the absence of other patterns of involvement may be explained by operative selection bias and the unavailability of PET-CT for PSL.

PSLs are best recognized on contrast-enhanced CT scans. Hypo-enhancing foci with diameters of less than 1 cm may be apparent after the administration of intravenous contrast material [13]. In cases with multiple masses, many small lesions less than 1 cm were clearly observed in addition to the large mass.

#### MRI findings

PSLs have a hypointense signal on T_1_WI and a varied signal appearance on T_2_WI that may be similar to that of normal splenic parenchyma, as demonstrated in our cases. On DWI, PSLs have a hyperintense signal, i.e., the lesion has a relatively lower apparent diffusion coefficient (ADC), which represents restriction of the free diffusibility of water molecules. This restriction likely occurs because PSLs have high cellularity and large abnormal nuclei and are therefore observed as a more densely packed tissue [18]. Gadolinium-enhanced sequences are more sensitive for the evaluation of splenic lymphoma, and focal disease can be observed as a hypointense focal lesion relative to the uniformly or arciform enhanced spleen [19]. Three cases in our series were evaluated using contrast-enhanced MRI; in these cases, imaging showed mild enhancement and hypointense signals relative to the clearly enhanced spleen.

#### Perisplenic involvement

Direct invasion may occur as noted in the 2 cases discussed above. Retroperitoneal lymph node and liver involvement represents the most common form of advanced stage disease. CT and MRI are excellent tools for the detection of lymphomatous involvement. Indeed, these imaging modalities are crucial for the clinical management and prognosis of such patients; data have shown that the survival rate of stage I patients is much better than that of stage III patients [20].

As a whole body imaging modality, PET-CT plays an important role in the staging, restaging and therapy monitoring of lymphoma [14]. This is primarily because PET-CT can detect ^18^F-FDG avid in normal-sized lymph nodes and distant involved sites that could be missed with CT or MRI. Unfortunately, no patients underwent PET-CT before surgery of the 17 patients enrolled because all patients included in this study showed no evidence of general lymphoma and could not be regarded as candidate for PET-CT for staging. Nevertheless, 2 patients in this group underwent a PET-CT exam after surgery, and no additional tumors were found, which confirmed the diagnosis of PSL.

Diffusion-weighted whole-body imaging with background body signal suppression (DWIBS) is a new diagnostic MRI sequence for staging malignant tumors. DWIBS seems to be a valuable technique for lymphoma staging, and its performance is equivalent to that of PET-CT [21]. The real impact of DWIBS remains to be determined by studies involving a larger series.

### Imaging diagnosis and differential diagnosis

The range and imaging similarity of splenic tumors explains the difficulty of obtaining accurate radiographic diagnoses of splenic lymphomas. CT alone has been reported to have an overall accuracy of 57% in diagnosing splenic involvement of lymphoma during initial staging [15], which is similar to the accuracy obtained in our study (47%, 8/17). In patients without peripheral adenopathy (stage I), the diagnosis may be even more likely to be overlooked. Indeed, in our report, the preoperative diagnostic accuracy of disease without evidence of peripheral adenopathy was 12% (1/8). However, imaging continues to be a mainstay for staging in this population.

The differential diagnosis of a solitary splenic mass should include benign entities, such as hemangioma, lymphangioma, hamartoma, inflammatory pseudotumor, infarct, abscess, and metastatic disease. In an asymptomatic patient, the most likely diagnosis of a focal splenic mass is hemangioma, with lymphangioma and hamartoma representing less likely possibilities. Hemangiomas usually demonstrate early peripheral nodular enhancement with progressive filling-in that is homogeneous on delayed scanning. Acute infarcts are always painful, and a predisposing cause may be clinically evident. In an afebrile, symptomatic patient, metastatic disease should be considered, and primary malignancy should be sought. If a primary malignancy is not found, then primary splenic lymphoma should be considered.

### Limitations

The limitations of this study relate to its retrospective nature and the relatively limited number of cases due to the rarity of PSL. In addition, despite the fact that there was strong evidence to confirm the diagnosis of PSL without involvement of a distant site, PET-CT or whole-body MRI data prior to surgery were not available in this study.

## Conclusion

PSL is a rare disease currently without a clear definition. The most frequent histologic type of this condition is DLBCL followed by SMZL. On CT and MRI, PSL generally presents as a solitary mass or masses rather than splenomegaly, and necrosis and mild enhancement are commonly observed. Findings of a splenic mass or splenomegaly in conjunction with splenic hilar adenopathy are suggestive of lymphoma, although splenectomy may be required for definitive diagnosis and histological subtyping.

## References

[1] Giovagnoni A, Giorgi C, Goteri G (2005) Tumours of the spleen. Cancer Imaging 5: 73–77. 16154823 10.1102/1470-7330.2005.0002 PMC1665244

[2] Ahmann DL, Kiely JM, Harrison EG Jr, Payne WS (1966) Malignant lymphoma of the spleen. A review of 49 cases in which the diagnosis was made at splenectomy. Cancer 19: 461–469. 5327041 10.1002/1097-0142(196604)19:4<461::aid-cncr2820190402>3.0.co;2-x

[3] Swerdlow SH, Campo E, Harris NL, Jaffe ES, Pileri SA, et al.. (2008) World Health Organization classification of tumors of haematopoietic and lymphoid tissues. lyon, France: IARC Press.

[4] Campo E, Swerdlow SH, Harris NL, Pileri S, Stein H, et al (2011) The 2008 WHO classification of lymphoid neoplasms and beyond: evolving concepts and practical applications. Blood 117: 5019–5032. 21300984 10.1182/blood-2011-01-293050 PMC3109529

[5] Dasgupta T, Coombes B, Brasfield RD (1965) Primary Malignant Neoplasms of the Spleen. Surg Gynecol Obstet 120: 947–960. 14269844

[6] Dachman AH, Buck JL, Krishnan J, Aguilera NS, Buetow PC (1998) Primary non-Hodgkin's splenic lymphoma. Clin Radiol 53: 137–142. 9502091 10.1016/s0009-9260(98)80061-5

[7] Kehoe J, Straus DJ (1988) Primary lymphoma of the spleen. Clinical features and outcome after splenectomy. Cancer 62: 1433–1438. 3416282 10.1002/1097-0142(19881001)62:7<1433::aid-cncr2820620731>3.0.co;2-v

[8] Kraemer BB, Osborne BM, Butler JJ (1984) Primary splenic presentation of malignant lymphoma and related disorders. A study of 49 cases. Cancer 54: 1606–1619. 6548171 10.1002/1097-0142(19841015)54:8<1606::aid-cncr2820540823>3.0.co;2-5

[9] Morel P, Dupriez B, Gosselin B, Fenaux P, Estienne MH, et al (1993) Role of early splenectomy in malignant lymphomas with prominent splenic involvement (primary lymphomas of the spleen). A study of 59 cases. Cancer 71: 207–215. 8416717 10.1002/1097-0142(19930101)71:1<207::aid-cncr2820710132>3.0.co;2-0

[10] Goh KO, Herrmann MA (1984) Abnormal chromosomes in histocytic lymphoma. Am J Med Sci 288: 48–52. 6380292 10.1097/00000441-198407000-00013

[11] Nakashima A, Nakashima K, Seto H, Kamei T, Kakishita M, et al (1994) Primary splenic lymphoma presenting as a large cyst. Radiat Med 12: 42–45. 8016404

[12] Fenchel S, Boll DT, Fleiter TR, Brambs HJ, Merkle EM (2003) Multislice helical CT of the pancreas and spleen. Eur J Radiol 45 Suppl 1: S59–72. 12598029 10.1016/s0720-048x(02)00363-7

[13] Leite NP, Kased N, Hanna RF, Brown MA, Pereira JM, et al (2007) Cross-sectional imaging of extranodal involvement in abdominopelvic lymphoproliferative malignancies. Radiographics 27: 1613–1634. 18025507 10.1148/rg.276065170

[14] Paes FM, Kalkanis DG, Sideras PA, Serafini AN (2010) FDG PET/CT of extranodal involvement in non-Hodgkin lymphoma and Hodgkin disease. Radiographics 30: 269–291. 20083598 10.1148/rg.301095088

[15] Rini JN, Leonidas JC, Tomas MB, Palestro CJ (2003) 18F-FDG PET versus CT for evaluating the spleen during initial staging of lymphoma. J Nucl Med 44: 1072–1074. 12843223

[16] Ambulkar I, Kulkarni B, Borges A, Jagannath P, Advani SH (2006) Primary non-Hodgkin's lymphoma of the spleen presenting as space occupying lesion: a case report and review of literature. Leuk Lymphoma 47: 135–139. 16321838 10.1080/10428190500277142

[17] Kirshtein B, Yelle JD, Moloo H, Poulin E (2008) Laparoscopic adrenalectomy for adrenal malignancy: a preliminary report comparing the short-term outcomes with open adrenalectomy. J Laparoendosc Adv Surg Tech A 18: 42–46. 18266573 10.1089/lap.2007.0085

[18] Humphries PD, Sebire NJ, Siegel MJ, Olsen OE (2007) Tumors in pediatric patients at diffusion-weighted MR imaging: apparent diffusion coefficient and tumor cellularity. Radiology 245: 848–854. 17951348 10.1148/radiol.2452061535

[19] Elsayes KM, Narra VR, Mukundan G, Lewis JS Jr, Menias CO, et al (2005) MR imaging of the spleen: spectrum of abnormalities. Radiographics 25: 967–982. 16009818 10.1148/rg.254045154

[20] Sumimura J, Miyata M, Nakao K, Kamiike W, Yamaguchi T, et al (1992) Primary malignant lymphoma of the spleen. Surg Today 22: 371–375. 1392349 10.1007/BF00308749

[21] Stephane V, Samuel B, Vincent D, Joelle G, Remy P, et al (2013) Comparison of PET-CT and magnetic resonance diffusion weighted imaging with body suppression (DWIBS) for initial staging of malignant lymphomas. Eur J Radiol In press. 10.1016/j.ejrad.2013.05.042 23932096

